# Mechanics of very slow human walking

**DOI:** 10.1038/s41598-019-54271-2

**Published:** 2019-12-02

**Authors:** Amy R. Wu, Cole S. Simpson, Edwin H. F. van Asseldonk, Herman van der Kooij, Auke J. Ijspeert

**Affiliations:** 1 Department of Mechanical and Materials Engineering, Queen’s University, Kingston, K7L 2V9 Canada; 2 Department of Mechanical Engineering, Stanford University, Stanford, CA USA; 3 Department of Biomechanical Engineering, University of Twente, Enschede, Netherlands; 4 Department of Biomechanical Engineering, Delft University of Technology, Delft, Netherlands; 5 Biorobotics Laboratory, École Polytechnique Fédérale de Lausanne, Lausanne, Switzerland

**Keywords:** Biomechanics, Mechanical engineering

## Abstract

Human walking speeds can be influenced by multiple factors, from energetic considerations to the time to reach a destination. Neurological deficits or lower-limb injuries can lead to slower walking speeds, and the recovery of able-bodied gait speed and behavior from impaired gait is considered an important rehabilitation goal. Because gait studies are typically performed at faster speeds, little normative data exists for very slow speeds (less than 0.6 ms${}^{-1}$). The purpose of our study was to investigate healthy gait mechanics at extremely slow walking speeds. We recorded kinematic and kinetic data from eight adult subjects walking at four slow speeds from 0.1 ms${}^{-1}$   to 0.6 ms${}^{-1}$   and at their self-selected speed. We found that known relations for spatiotemporal and work measures are still valid at very slow speeds. Trends derived from slow speeds largely provided reasonable estimates of gait measures at self-selected speeds. Our study helps enable valuable comparisons between able-bodied and impaired gait, including which pathological behaviors can be attributed to slow speeds and which to gait deficits. We also provide a slow walking dataset, which may serve as normative data for clinical evaluations and gait rehabilitative devices.

## Introduction

Healthy humans walk at various speeds and can perform a multitude of speed adjustments throughout a day in response to environmental factors, but the ability to regulate speed can diminish with age, neurological deficits, or injuries. Ambulatory individuals with spinal cord injuries may be limited to 0.20–0.88 ms${}^{-1}$ ^1–3^, stroke survivors to 0.23–0.73 ms${}^{-1}$ ^4^, and children with cerebral palsy to 0.02–0.36 ms${}^{-1}$ ^5^. The range of walking speeds for these populations depends on the severity of their condition and any recovery from gait training programs, but the upper limits are still considerably slower than the preferred walking speeds of healthy individuals (1.20 ms${}^{-1}$  to 1.40 ms${}^{-1}$)^6,7^. In older adults, walking speed is associated with functional capability, physical and cognitive decline, fall risk, and median life expectancy^8–10^. However, healthy data is mainly centered around walking speeds faster than those of elderly or impaired individuals^11–13^. This lack of normative data on healthy gait mechanics at extremely slow speeds may limit assessments of impaired behavior and hinder gait recovery efforts.

Healthy gait behaviors at speeds between 0.6 ms${}^{-1}$  and the walk-run transition of 2.0 ms${}^{-1}$  are more commonly measured than speeds below 0.6 ms${}^{-1}$ ^12,14–16^. At these faster speed ranges, gait mechanics typically scale with speed. For example, body center of mass (COM) and joint kinematic and kinetic measures exhibit larger magnitudes at faster speeds^14,15,17^. Several relationships between various spatiotemporal and mechanical factors have also been established empirically or from simple models of walking^18,19^. In contrast, very few studies have investigated gait changes of healthy subjects at speeds slower than 0.6 ms${}^{-1}$. Kinematic data has been recorded at speeds as low as 0.14 ms${}^{-1}$ ^1,20^, 0.2 ms${}^{-1}$ ^21,22^, and 0.28 ms${}^{-1}$ ^23^. van Hedel *et al*., who recorded both gait kinematics and electromyographic (EMG) data, reported that muscle activity and joint kinematics changed at speeds below 0.69 ms${}^{-1}$, when they were more poorly correlated to 1.4 ms${}^{-1}$  reference speed^1^. den Otter *et al*., who only recorded EMG data from speeds as low as 0.06 ms${}^{-1}$, found that the peroneus longus activity was positively correlated with decreased speeds, indicating a need for lateral balance control^24^. Only two studies have reported kinetic data, including ground reaction forces and moments^25^ and joint moments and powers^23^. None reported mechanical work measures, such as joint work and work done on the body center of mass (COM), or evaluated them in the context of known relationships established at faster speeds.

It is unclear whether very slow walking will be markedly different from normal walking. As speed slows, kinematic range of motion reduces^15^, and stance times increase^21,26^. The reduction in dynamic behavior combined with the need to maintain balance over longer periods of time could produce more variable stepping behavior. However, some studies suggest that slow walking is more stable than fast walking^27,28^. Then, despite variations in behavior, slow walking could be simply a scaled version of preferred walking. Known relationships for spatiotemporal and work measures with respect to speed could still hold because the dynamic walking models^19,29^ on which they are based are still valid.

The purpose of our study was to investigate normative gait kinematics and kinetics at extremely slow walking speeds of 0.1 ms${}^{-1}$  to 0.6 ms${}^{-1}$. We hypothesize that speed-related changes at slow speeds will be consistent with those reported at faster speeds. Our analysis is mainly restricted to the sagittal plane, where dynamic walking models have demonstrated that gait is passively stable^19,29^. Since human gait in the sagittal plane can be stabilized by passive dynamics with little neural feedback control^30^, predictions from dynamic walking models for mechanics-related changes, such as step length and joint work, should hold for all non-zero walking speeds. Therefore, we expect that gait behaviors during very slow walking will not be considerably different from behaviors at faster speeds. As we will see next, the slow walking data from this study, which is provided in a public repository (see Data Availability), supports that prediction.

## Results

We observed several changes in gait mechanics at very slow speeds, but none that were markedly different from speed-related changes at faster walking speeds. As speed decreased, subjects spent more time in stance but took shorter steps. Step length (and step time) vary strongly with speed, but changes in step width or step variability were either minor or insignificant. Ground reaction force, COM power, and summed joint power magnitudes all decreased with speed, along with magnitudes of joint angles, torques, and powers. COM and summed joint work rates decreased linearly with speed, and COM work during collision and push-off decreased in magnitude in proportion to ${v}^{2.8}$. Trends from fits performed with respect to speed are summarized in Table 1.

**Table 1:** Quantitative results for fits to gait measures with respect to speed. Fit parameters include trend value (means $\pm$ 95% confidence interval, CI) and offsets (means $\pm$ s.d.). Linear fits to speed were performed for all measures except for known nonlinear relations for step length, step period, double support period, and COM work phases (collision, rebound, reload, and push-off). ${R}^{2}$ values indicate the goodness of fit, and P-values indicate statistical significance of the trend (*$P < 0.05$). Fits were performed on normalized data. Quantities are reported in dimensionless form, with body mass, gravitational acceleration and leg length as base variables.

Gait measure	Coefficient (mean $\pm$ CI)	Offset (mean $\pm$ s.d.)	${R}^{2}$	$P$
Step Length	0.577 $\pm$ 0.061	1.243 $\pm$ 1.135	0.949	7.25e-16*
Step Width	0.068 $\pm$ 0.069	0.132 $\pm$ 0.065	0.974	5.27e-02
Step Length RMS	0.007 $\pm$ 0.028	0.017 $\pm$ 0.006	0.661	6.08e-01
Step Width RMS	0.049 $\pm$ 0.012	0.010 $\pm$ 0.005	0.921	9.11e-09*
Step Period	$-0.423\pm 0.061$	1.243 $\pm$ 1.135	0.914	5.40e-13*
Double Support Period	$-0.721\pm 0.084$	0.257 $\pm$ 1.159	0.937	6.15e-15*
COM Work Rate (pos)	0.031 $\pm$ 0.003	0.001 $\pm$ 0.001	0.946	2.29e-15*
COM Work Rate (neg)	$-0.027\pm 0.003$	$-0.001\pm 0.001$	0.940	4.52e-14*
Summed Joint Work Rate (pos)	0.066 $\pm$ 0.006	$-0.000\pm 0.002$	0.962	1.11e-16*
Summed Joint Work Rate (neg)	$-0.053\pm 0.006$	$-0.001\pm 0.002$	0.940	2.11e-14*
Collision	$-0.174\pm 0.109$	$-0.001\pm 0.002$	0.896	4.51e-03*
Rebound	$-0.015\pm 0.114$	0.003 $\pm$ 0.001	0.704	7.76e-01
Preload	$-0.383\pm 0.178$	$-0.007\pm 0.003$	0.889	5.27e-04*
Push-off	0.507 $\pm$ 0.141	0.008 $\pm$ 0.005	0.966	4.60e-06*

**Figure 1:**
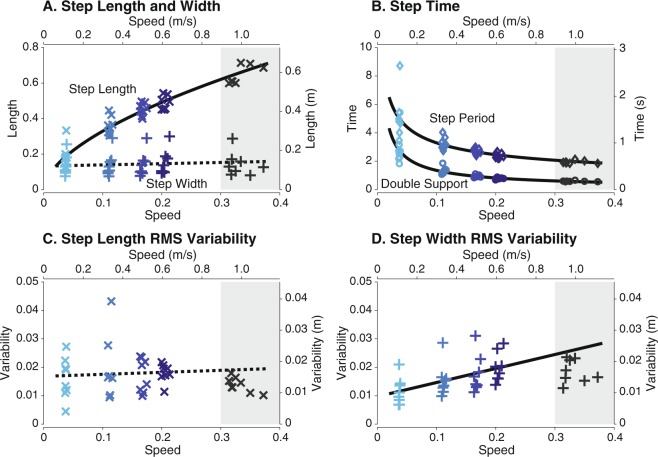
Mean step parameters over a range of speeds. As speed decreased, (**A**) step length decreased with no significant changes in step width, (**B**) step period and double support period increased, (**C**) no significant change was found for step length variability, and (**D**) step width variability decreased. Variability is defined as root-mean-square (RMS) deviations from mean steps. Trend significance indicated by solid lines ($P < 0.05$) and non-significance by dashed lines. Data at self-selected speeds not included in fit (shaded region). Vertical axes are shown in both dimensionless (left axes) and SI form (right axes); speed in horizontal axes are also shown in both dimensionless (lower axes) and SI form (upper axes).

We found that step length, step period, double support period, and step width variability changed with speed but not step width or step length variability (Fig. 1). We fit step length with $l\propto {v}^{\beta }$ and found that the relationship is still valid at slow walking speeds with $\beta =0.577\pm 0.061$ (mean $\pm \,95$% confidence interval, CI, $P$ = 7.25e-16, ${R}^{2}=0.949$, Fig. 1A). In contrast to step length, step width did not change significantly with speed ($P=0.053$, Fig. 1A). Step time and double support time both increased nonlinearly as speed decreased (Fig. 1B). Fitting the same power relation as step length for step time yielded a coefficient of $-0.423\pm 0.061$ (mean $\pm$ CI, ${R}^{2}$ = 0.914, $P$ = 5.40e-13, Fig. 1B). Step length variability did not significantly change with speed ($P$ = 0.608, Fig. 1C). Step width variability decreased as speed slowed at an average rate of 15 mm per ms${}^{-1}$  ($P$ = 9.11e-09, Fig. 1D). The significant step parameter trends predicted values near self-selected, except for the overestimation in step width variability.

**Figure 2:**
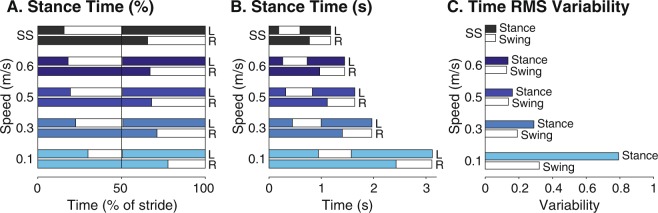
Mean stance and swing times across speeds. Mean stance time (colored bar) of a gait cycle for left (L) and right (R) legs as (**A**) percentage of stride and (**B**) time in seconds. As speed decreased, the percentage of single support time (white bar) decreased and double support time increased (overlap of colored bars), leading to an increase in stance phase overall. While single support decreased as a percentage of stride, single support increased in time due to longer stride times. (**C**) Variability in both stance time and swing time increased as speed slowed. Variability is defined as RMS deviations from mean times and shown in dimensionless units. Data shown were averaged across all subjects (N = 8).

Stance time increased with slower speeds due to more time spent in the double support phase than the single support phase (Fig. 2). Subjects spent 78% $\pm$ 2.2% (mean $\pm$ s.d.) of the gait cycle in stance at the slowest speed, 19% more than at self-selected speeds (Fig. 2A). At the slowest speed, double support and single support phases contribute to 55% and 21% of the gait cycle, compared to 12% and 35% at self-selected speeds, representing a 4.5 times increase and 1.7 times decrease, respectively. In time units, the lengthening of double support duration still dominated over single support duration. Double support duration increased by nearly five times, while single support time duration only increased by 1.5 times from self-selected to slowest speeds (Fig. 2B). Variability in stance and swing time also increased at slow speeds, with larger variations for stance than swing times (Fig. 2C).

**Figure 3:**
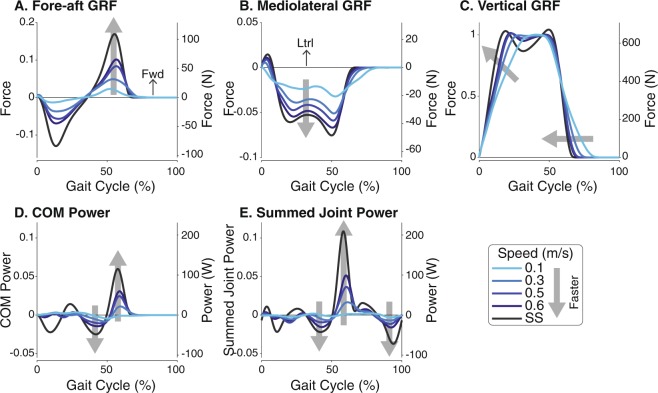
Mean force and power measures as a percentage of stride over a range of speeds. (**A**) Fore-aft, (**B**) mediolateral, and (**C**) vertical ground reaction forces (GRFs), (**D**) COM power, and (**E**) summed joint power decreased in magnitude as speed decreased. Vertical axes are shown in both dimensionless (left axes) and SI form (right axes); horizontal axes are shown as a fraction of gait cycle (% of stride) beginning with heel-strike. Data shown were averaged across all subjects (N = 8).

**Figure 4:**
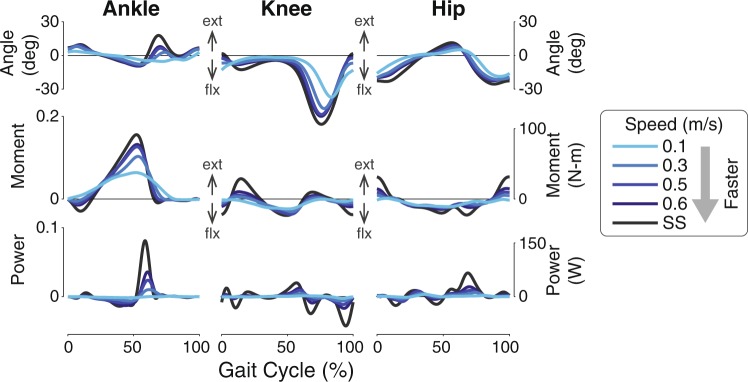
Mean joint angle, moment, and power in the sagittal plane as a function of gait cycle. Left-hand axes for moment and power are in dimensionless units, and right-hand axes are SI units. Horizontal axes are shown as a fraction of gait cycle (% of stride) beginning with heel-strike. Ext: extension (plantarflexion), flx: flexion (dorsiflexion). Data shown were averaged across all subjects (N = 8).

Slow walking speeds tend to scale down and flatten ground reaction forces (Fig. 3A–C). Peak fore-aft forces decreased with slow speeds, and medial forces also decreased. For the vertical forces, the loading and unloading slopes became shallower, indicating a slow transfer of forces from one leg to another (i.e. longer double support duration). The double peak characteristic of typical walking speeds was also attenuated. While slow speeds elongated the mediolateral and vertical forces in duration, the fore-aft forces are nearly zero after approximately 63% of the gait cycle. This suggests that fore-aft dynamics are affected in magnitude but not timing, which could partially be attributed to passive stability in the sagittal plane. The scaling of forces were also seen in COM power (Fig. 3D) and summed joint power (Fig. 3E). Both positive and negative power magnitudes decreased with slower speeds, especially between 40% and 60% of stride. Unlike ground reaction forces, the power curves at the slowest speed are visually dissimilar from the power patterns at faster speeds. The observed changes in joint angles, moments, and powers were also scaled in magnitude with speed (Fig. 4), trending towards the zero as speed decreased. Similar to COM and summed joint powers, the diminished magnitudes are most apparent near push-off.

**Figure 5:**
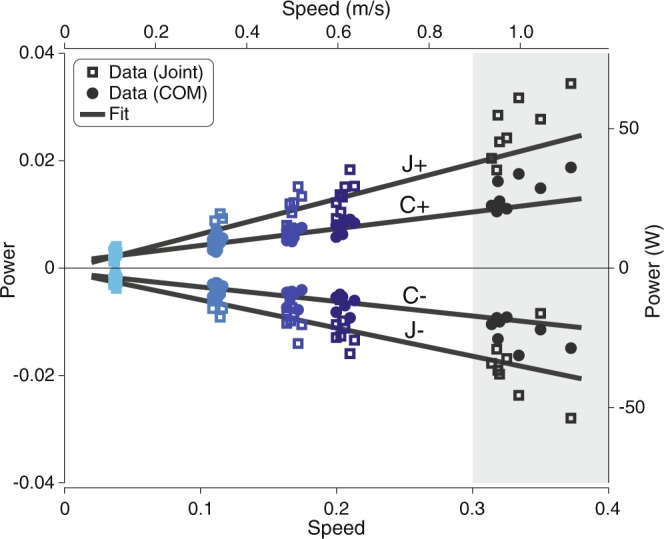
Mean mechanical work rates across walking speeds. Mean COM work rate per stride (C+, ${\rm{C}}-$) and mean summed joint work rate per stride (J+, ${\rm{J}}-$) decreased linearly with speed, with a steeper rate of decrease for the summed joint work rates. Different colors denote subjects’ data at each walking speed (squares for joint work rates, circles for COM work rates). Trend significance is indicated by solid lines ($P < 0.05$). Data at self-selected speeds not included in fit (shaded region). Power is shown in both dimensionless (left axis) and SI form (right axis); speed is also shown in both dimensionless (lower axis) and SI form (upper axis).

Qualitative observations of force and power measures of the COM and summed joint are supported by quantitative changes in the mechanical work performed by the body. COM work rates and summed joint work rates decreased linearly as speed decreased (Fig. 5). Positive work rates decreased about 68% (COM) and 83% (joint) from the fastest prescribed speed (0.6 ms${}^{-1}$) to the slowest (0.1 ms${}^{-1}$). Similar values were found for negative work rates. Fit predictions of work rates at self-selected speeds seem aligned with subject data (18% error for positive work rates of both measures).

**Figure 6:**
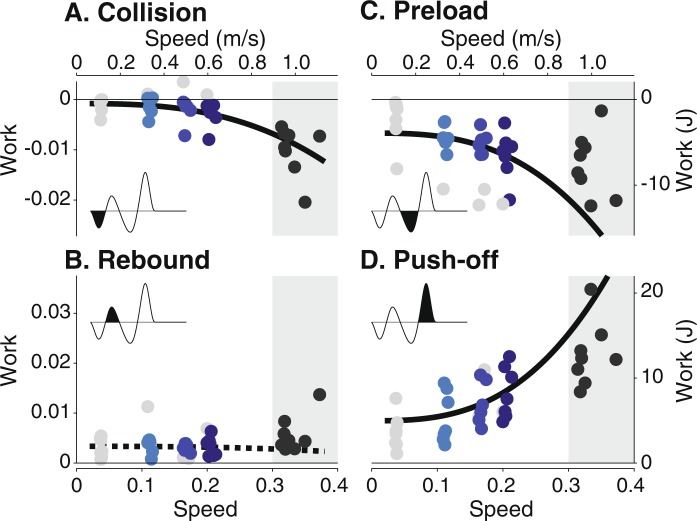
Mean COM work against walking speed for each phase of the gait cycle. The phases are (**A**) collision, (**B**) rebound, (**C**) preload, and (**D**) push-off. Fits were performed with COM work as a function of ${v}^{2.8}$ based on dynamic walking models^15^. Three of four (collision, preload, push-off) decreased nonlinearly in magnitude as speed decreased. Different colors denote subjects’ data at each walking speed (circles). Trend significance is indicated by solid lines ($P < 0.05$) and non-significance by dashed lines. COM work phases were less distinguishable at slower speeds. Trials with phases detected for less than 50% of the strides were not included in the fit (denoted by grey symbols). Data at self-selected speeds not included in fit (shaded region). Work is shown in both dimensionless (left axes) and SI form (right axes); speed is also shown in both dimensionless (lower axes) and SI form (upper axes).

Work decreased in magnitude with ${v}^{2.8}$ as speed decreased for three of the four phases of COM work (Fig. 6). Both collision work and push-off work reduced in magnitude ($P$ = 4.51e-3 and $P$ = 4.60e-6) as speed decreased, with collision work nearing zero. Preload work also decreased with speed ($P$ = 5.27e-4). No significant trend was found during rebound ($P$ = 0.776). COM work predictions matched roughly with self-selected data for the collision and push-off phases but not for the preload phase. The preload trend estimated a greater decrease in work than actually performed (208% error, compared with 33% and 56% error for collision and push-off). COM work phases became less distinguishable at slow speeds, with approximately 12 out of 32 trials excluded from the fits for this measure, including all trials at the slowest speed. Of the included slow walking trials, phase timing did not change significantly for collision (mean 19% of stride at end of phase) or preload (mean 52% of stride). End of phase timing for rebound and push-off decrease slightly with faster speeds from an average of 34% to 30% and 69% to 67%, respectively.

## Discussion

Our study sought to evaluate whether speed-related trends at slower speeds are comparable to established trends at faster speeds. We observed several changes in gait mechanics at very slow speeds, including shorter step lengths, longer step times, and smaller force and power magnitudes. We performed fits of gait measures with respect to speed to determine how well known relations at faster speeds can predict trends at much slower speeds. Much of the qualitative and quantitative kinematic and kinetic changes are consistent with previous studies at faster speed ranges^1,6,20,23,26,31^. Except for step width variability and COM work during preload, trends derived from slow speeds provided reasonable estimates of gait measures at self-selected speeds.

The power law that governs the preferred step length and speed relation was still valid at very slow speeds. To walk faster, one can either adjust their step length, step frequency, or a combination of both. Increases in step length can be costly due to greater losses at collision^32^, and increases in step frequency can also be costly due to the production of force over short periods of time to generate leg swing^33^. Empirical data and walking models have shown that the power law coincides with the most economical combination of step length and step frequency at given walking speeds^19,34^. We found a $\beta$ coefficient within range of previous results^18,35^, demonstrating that the energetic trade-offs that affect walking at faster speeds are still important at slow speeds. These results seem to contradict a previous study on the walk ratio, the ratio of step length to step frequency, which suggested that energy optimization is not maintained at speeds slower than 1 ms${}^{-1}$ ^36^. The calculation of the walk ratio from the power law (walk ratio $\propto {v}^{2\beta -1}$) with our $\beta$ coefficient yields a small power term that produces a near constant walk ratio, especially at faster walking speed ranges. This behavior suggests that our power law results are consistent with previous findings of a near constant walk ratio, despite our differing interpretations of energetic economy.

Other observed spatiotemporal changes with speed agree with those reported in literature. Previous studies have also found that stance time, as a percentage of stride, increased with slower speeds^21,26^ with a reduction in single support duration and an increase in double support^6^. The greater variations in stance time than swing time has also been reported^37^. We found that step width did not significantly change with speed (see Fig. 1A). Our results agree with existing lack of consensus whether step width is affected by speed. One study showed that stride width increased with decreased speed^14^ while another showed no speed dependence^35^. While these differences could be related to overground versus treadmill walking, the relationship between step width and speed seems tenuous.

Quantitative relationships between COM and summed joint work and work rates generally agree with previous studies^15,17^. We found that ${v}^{2.8}$ was a good predictor of COM work during collision (${R}^{2}=0.896$) and push-off (${R}^{2}=0.966$). Comparing prediction errors at self-selected speeds, the fit of collision work at very slow speeds was the strongest predictor for faster speeds over the other three phases of COM work. Energy losses around heelstrike therefore seem to change consistently across speeds, likely due to step length changes. The addition of positive work through push-off to compensate for negative work was also a fairly good estimator of work at faster speeds. However, preload work at slow speeds was a poor indicator. We found that preload decreased with faster speeds, in contrast to others who reported a slight increase in preload work^15^. We did not expect a linear relationship between work rates and speed based on simple models of walking^17^. Converting $W\propto {v}^{2.8}$ to work rates yielded $\dot{W}\propto {v}^{3.4}$. However, linear fits with respect to speed performed better (${R}^{2}$ from 0.940 to 0.962) than fits to speed powered (${R}^{2}$ from 0.857 to 0.891). The overall consistent behavior in joint work and work rates across speeds may be supported by a previous study that found no change in kinematic and kinetic parameters from speeds between 0.2 ms${}^{-1}$ and 0.8 ms${}^{-1}$ ^22^.

There could be differences in speed-related gait behavior among subjects. Our fits allowed individual offsets for each subject but only one trend for all subjects’ data. The ${R}^{2}$ values (see Table 1) suggest a high goodness-of-fit for most measures and little inter-subject variability in trends across speeds. Differences among subjects could also amplify at slower speeds. Visual inspection of step time and double support time subject data (see Fig. 1B) reveal more inter-subject variability at slower speeds. The standard deviation of mean subject step times was 8.4 times larger at 0.1 ms${}^{-1}$  than at 0.6 ms${}^{-1}$. Step length and step width do not exhibit similar variations in behavior, suggesting that step placement was fairly consistent among subjects at all speeds while step timing was not.

One limitation of our study is that we did not obtain data at faster walking speeds. We cannot directly compare regressions between very slow and fast speeds or determine the existence of a speed inflection point that differentiates gait behaviors between slower and faster speeds^22,37^. Instead, we evaluated slow walking regression parameters with their estimate of gait measures at self-selected speeds of maximum 0.99 ms${}^{-1}$ on average. Although we cannot conclude from our data whether the coefficients from our regression are still applicable at faster walking speeds, we believe they are still valid at fast speeds for some measures. Our $\beta$ coefficient (0.577 $\pm$ 0.061, mean $\pm$ CI) is similar to reported coefficients (0.54 $\pm$ 0.10) from regressions performed at walking speeds up to 1.8 ms${}^{-1}$^35^. Our coefficient for COM work during collision ($-0.174\pm 0.109$) is also similar to those obtained at speeds between 0.7 ms${}^{-1}$  and 2.0 ms${}^{-1}$  ($-0.178\pm 0.014$)^15^. However, for COM work during push-off, our coefficient (0.507 $\pm$ 0.141) predicted work that was much greater than the work done at faster speeds (0.095 $\pm$ 0.012)^15^.

The implications of our results for neural control of human gait are unclear. The increased variability in stance times suggests stance and swing behaviors are generated by differing mechanisms^38^. Possible reasons for the relatively consistent swing time behavior include the utilization of natural swing dynamics^33^ or some swing generator-like mechanism^39^. In contrast, the stance phase could be governed by feedback from load receptors^39^ or active control mechanisms during double support^40^ or single stance^41^, leading to increased variations in behavior. As speed changes, gait kinematics and kinetics seem to scale along a continuum rather than discretely, which could be interpreted as evidence of speed modulation through central pattern generators^42^ or adjustment of reflex gains^43^. However, very slow speeds have proven difficult to produce in both neural oscillator and reflex-based models^43–45^, with a typical lower speed bound of 0.7 ms${}^{-1}$. These challenges suggest a need for more biologically accurate simulation models or the existence of a new control regime or discrete change that is currently not identifiable in our results.

We primarily evaluated gait behavior in the sagittal plane, but gait behavior in the frontal plane could show more deviations between very slow and faster speeds, potentially allowing greater insight into neural control mechanisms. Stability in the frontal plane seems to decrease with speed^46^, and unlike the sagittal plane, the frontal plane requires active control for stabilization^30^. Therefore, mediolateral gait behaviors could be considerably different at very slow speeds. As a future area of study, we plan to evaluate control strategies to determine how subjects maintain balance at very slow speeds and at their self-selected speed.

In addition to investigating slow walking data, our goal was to provide normative very slow walking data. Healthy adult walking data is typically captured at walking speeds 0.6 ms${}^{-1}$, or greater, and without comparable slow speed data, the evaluation of the slower gait of neurologically or functionally impaired individuals is difficult. We did not prescribe speeds slower than 0.1 ms${}^{-1}$  because they were impractical to perform on a treadmill. We have deposited all slow walking data necessary to reproduce our results in a openly accessible repository (see Data Availability section). Our dataset contributes both kinematic and kinetic data and derived work measures. Although our observations were obtained over a limited, homogeneous population of healthy adults, walking speed seems to influence gait kinematics more than age for subjects within 19 and 67 years of age^47^. Our data augments existing datasets at faster speeds, enabling the research community to access normal walking data at slow extreme of the speed spectrum.

With a comprehensive investigation of gait mechanics at very slow speeds, the insights from our study could help determine stability issues or compensatory strategies that are inherent to slow walking, which are not necessarily captured at typical speeds reserved for healthy subjects. Our normative slow walking data will also help differentiate among difficulties experienced by impaired walkers into those that are a consequence of very slow walking speeds and of the person’s pathological condition. Recognizing differences between normal and abnormal slow walking will aid the design of new gait rehabilitation training programs and controllers for assistive technologies.

## Methods

To determine the mechanics of walking at very slow speeds, we asked healthy, adult subjects to walk on an instrumented treadmill at four different slow walking speeds and one self-selected speed. We recorded ground reaction forces, kinematic data, and electromyographic data. A total of ten subjects participated in the study. Data from two subjects were removed due to equipment failure. The prescribed treadmill speeds were 0.4, 1.2, 1.8, and 2.2 km/h, termed 0.1, 0.3, 0.5, 0.6 ms${}^{-1}$, respectively. Self-selected speed of the reported subjects ($N$ = 8, six female, two male) ranged from 0.92 ms${}^{-1}$  to 1.14 ms${}^{-1}$. Subjects’ age ranged from 23 to 31 years. Each walking trial was two minutes long and performed in a randomized order. Their body mass $m$ was 65.6 $\pm$ 9.62 kg (mean $\pm$ s.d), and their leg length $L$ was 0.908 $\pm$ 0.041 m. All subjects provided written informed consent in accordance with the Declaration of Helsinki. The protocol was approved by the Medisch Ethische ToetsingsCommissie (METC) Twente.

Gait kinematics and kinetics were recorded to determine mechanics-related changes. We measured gait mechanics with motion capture (Visualeyez, Phoenix Technologies, Burnaby, Canada) and an instrumented treadmill (Motekforce Link, Amsterdam, the Netherlands). Marker data was acquired at 100 Hz, and force data at 2000 Hz. For the collection of kinematic data, we placed nine frames consisting of three active markers each on the feet, shank, thigh, pelvis, sternum, and head. Additional markers were placed on the lateral epicondyle of the femur and on the lateral malleolus. Prior to the experiment, bony landmarks were identified using marker probes^48^. Standard kinematic and inverse dynamic procedures were used in OpenSim^49^ to calculate spatiotemporal gait parameters, joint angles, and joint torques, similar to the process outlined in a previous study^41^. Briefly, marker trajectories and analog force and moment data were filtered using a fourth order, zero-phase Butterworth low-pass filter at 20 Hz. Force data was also resampled to 100 Hz to match marker data. Inverse kinematics and inverse dynamics were performed on the 23 degrees-of-freedom gait2345 OpenSim model. The resulting joint kinematics and kinetics, along with the ground reaction forces, were then filtered with a fourth-order, zero-phase 6 Hz Butterworth low-pass filter.

We also quantified power, work, and work rates. Joint power was calculated using joint angular velocities and joint torques. We defined summed joint power as the net power from the summation of ankle, knee, and hip power during each stride. We also measured the instantaneous COM work rate^32^, the rate of work performed on the body COM. The COM work rate was computed as the inner product of the 3D vectors of the ground reaction forces of each leg and the body COM velocity. The positive intervals of summed joint power and COM work rate were integrated to yield positive COM work and summed joint work per stride. The average positive summed joint and COM work rates for both sides of the body were calculated from dividing the positive work per stride by stride time and multiplying by 2. The same calculations were performed for negative work rates. COM work rates were also further divided into four phases that represent positive and negative intervals of collision, rebound, preload, and push-off and integrated to yield work (see dark shaded regions in exemplar COM work rate curve in Fig. 6).

We made qualitative observations of gait trajectories and summarized overall gait behavior through quantitative fits of mechanical measures. Gait trajectories (e.g. ground reaction forces, joint angles) are expected to scale with speed, and decreased magnitudes correspond to decreases in both positive and negative values. We quantified speed-related changes to gait mechanics through fits between speed and calculated measures. All data except at self-selected speeds were included in the fit. As the simplest model, linear fits were performed on all measures except those with known nonlinear relationships with speed. Empirical studies have shown that step length $l$ and speed $v$ are related through a power law, $l\propto {v}^{\beta }$, with $\beta$ ranging between 0.45 and 0.73 for walking speeds from 0.5 ms${}^{-1}$ to over 2.0 ms${}^{-1}$ ^18,35^. The power coefficient $\beta$ was calculated from the linear regression over the logarithm of subject data. The COM work for each phase also varies nonlinearly with speed. Dynamic walking models predict that COM collision work $W$ increases with speed raised to the power of 2.8 ($W\propto {v}^{2.8}$)^15,19^. The COM push-off work largely serves to offset the collision, likely increasing at similar rates with faster speeds^50,51^. COM work phases were less identifiable at slower speeds. Therefore, fits did not include trials with phases detected for less than 50% of the strides. Fits were performed on all subject data simultaneously, allowing one trend per measure and each subject to have an individual constant offset. Statistical tests were performed on the regression coefficients using the t-statistic with a significance level of $\alpha =0.05$.

Unless otherwise indicated, data have been non-dimensionalized by body mass $M$, leg length $L$, and gravity $g$. Step length and width were divided by $L$ (mean 0.908 $m$), step time by $\sqrt{L/g}$ (mean 0.304 $s$), walking speed by $\sqrt{gL}$ (mean 2.98 ms${}^{-1}$), force by $Mg$ (mean 644 $N$), torque and work by $MgL$ (mean 587 $Nm$), and power and work rate by ${Mg}^{1.5}{L}^{0.5}$ (mean 1926 $W$). The data analyzed in this study is openly accessible in a public repository (see Data Availability section).

## Data Availability

The datasets generated and analyzed during the current study are available in Queen's University Dataverse repository at 10.5683/SP2/EMQLLE.
